# Direct capture and conversion of CO_2_ from air by growing a cyanobacterial consortium at pH up to 11.2

**DOI:** 10.1002/bit.26974

**Published:** 2019-04-08

**Authors:** Maryam Ataeian, Yihua Liu, Karen Andrea Canon‐Rubio, Michael Nightingale, Marc Strous, Agasteswar Vadlamani

**Affiliations:** ^1^ Department of Geoscience University of Calgary Calgary Alberta Canada

**Keywords:** alkalinity, BECCS, Cyanobacteria consortium, direct carbon capture, photosynthesis, soda Lakes

## Abstract

Bioenergy with carbon capture and storage (BECCS) is recognized as a potential negative emission technology, needed to keep global warming within safe limits. With current technologies, large‐scale implementation of BECCS would compromise food production. Bioenergy derived from phototrophic microorganisms, with direct capture of CO_2_ from air, could overcome this challenge and become a sustainable way to realize BECCS.

Here we present an alkaline capture and conversion system that combines high atmospheric CO_2_ transfer rates with high and robust phototrophic biomass productivity (15.2 ± 1.0 g/m ^2^/d). The system is based on a cyanobacterial consortium, that grows at high alkalinity (0.5 mol/L) and a pH swing between 10.4 and 11.2 during growth and harvest cycles.

## 1 INTRODUCTION

Bioenergy with carbon capture and storage (BECCS) is the biological air‐capture and conversion of carbon dioxide, followed by biomass combustion and geological sequestration of carbon dioxide from the resulting flue gas. The technical, economical and environmental feasibility of negative emission technologies (NET) (Bui et al., 2018), such as BECCS, are currently under debate (Scott & Geden, 2018; Van Vuuren, Hof, Van Sluisveld, & Riahi, 2017; Xenias & Whitmarsh, 2018). For example, BECCS would require a large amount of arable land (~ 990 Mha; Bui et al., 2018), competing with food production (Ingram et al., 2015). To overcome this problem, several studies have proposed the use of biofuels/bioenergy derived from phototrophic microorganisms (instead of plants) as a means to simultaneously displace fossil fuels and capture carbon dioxide from the atmosphere (Beal, Archibald, Huntley, Greene, & Johnson, 2018; Vadlamani, Pendyala, Viamajala, & Varanasi, 2019; Wijffels & Barbosa, 2010). Growth of algae and cyanobacteria does not require arable land and the productivity (ton biomass per hectare per year) of algal growth systems can be higher than for typical energy crops such as palm oil and switch grass (Li, Horsman, Wu, Lan, & Dubois‐calero, 2008; Mata, Martins, & Caetano, 2010; Quinn, Catton, Wagner, & Bradley, 2012; Thomas‐Hall et al., 2008).

Algae and cyanobacteria cultivators typically feed carbon dioxide to growth systems by sparging or bubbling. However, with only 400 ppm carbon dioxide in air, such direct capture of CO_2_ from air would lead to excessive energy costs and evaporation (Davis, Aden, & Pienkos, 2011). To overcome this problem, carbon dioxide could first be captured from air and concentrated, for example by calcium looping (Keith, Holmes, St. Angelo, & Heidel, 2018). Alternatively, as previously proposed, we could avoid an energy‐expensive concentration step by using growth media with high pH and alkalinity. The ability of such media to dissolve a large amount of carbon dioxide as bicarbonate, would also enable implementation of CO_2_ capture and growth as two separate unit operations (Sharp, Canon‐Rubio, Strous, Bergerson, & De la Hoz Siegler, 2015; van Loosdrecht, Daelman, Strous, Sorokin, & Kruse, 2016), saving costs and energy. During CO_2_ capture from air, carbon dioxide first reacts with a hydroxide ion (OH^−^), yielding bicarbonate. The hydroxide ion is then regenerated by transfer of a proton from water to carbonate, yielding another bicarbonate ion, and leading to the overall equation:

(1) $${{\text{CO}}_{3}}^{2-}+{\text{CO}}_{2}\;+H_{2}O\;={{\text{2HCO}}_{3}}^{-}$$

**Figure 1:**
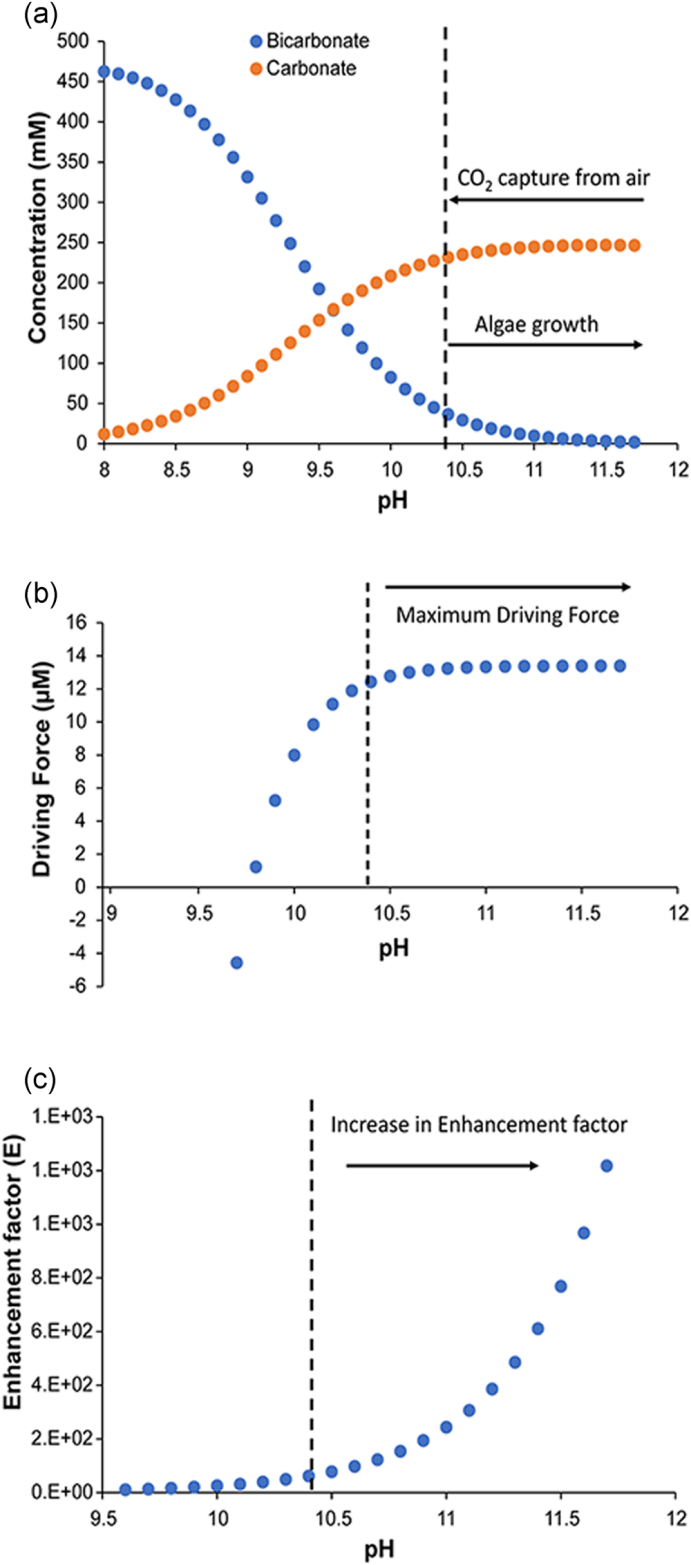
pH dependent changes in (a) HCO_3_^−^ and CO_3_^2−^ concentrations, (b) mass transfer driving, and (c) enhancement factor. The values were calculated for a cultivation medium with an alkalinity of 500 meq/L using pK_1_ and pK_2_ at temperature (T) 25°C and ionic strength (I) 0.73 mole/L. The dotted line in all panels indicates the lower bound of pH window compatible with both effective CO_2_ capture and effective algal growth (HCO_3_^−^ concentrations 30–40 mM at the start of the growth cycle) [Color figure can be viewed at wileyonlinelibrary.com]

During air capture, this equation leads to a decrease in pH (Figure 1a). However, it is important to understand that the RuBisCO enzyme that carries out photosynthetic carbon fixation converts CO_2_ and not HCO_3_^‐^. At high pH (>9), HCO_3_^−^ is the predominant species and the CO_2_ concentration is very low. To overcome the CO_2_ limitation posed by pH driven carbon speciation, algae and cyanobacteria have evolved carbon concentrating mechanisms (CCM's; Beardall & Raven, 1981; Raven, Cockell, & De La Rocha, 2008). These actively transport HCO_3_^−^ from the growth medium into the cell. Next, the HCO_3_^−^ inside the cells is converted to CO_2_, by carbonic anhydrase, for fixation by RuBisCO. To maintain electroneutrality within the cell, cells release OH^−^ into the external medium. This results in an overall increase in the medium pH:

(2) $${{\text{2HCO}}_{3}}^{-}={{\text{CO}}_{3}}^{2-}+{\text{CO}}_{2}\;+H_{2}O$$

For effective CO_2_ capture, the carbon dioxide concentration in the capture medium must be sufficiently low, because, the driving force for carbon dioxide transfer from air to medium equals the difference between the carbon dioxide concentration at the air/medium interface and its concentration in the remainder (bulk) of the medium. The former can be estimated to be approximately 13 µmol/L, using Henry's law constant with 400 ppm carbon dioxide in air and at 25°C. The bulk concentration is a function of the pK values of the carbonate buffer system and the bicarbonate concentration. Figure 1a shows the change in bicarbonate/carbonate speciation for a medium alkalinity of 0.5 M and over a pH range of 8−12. The estimated equilibrium pH is around 9.7. Figure 1b shows the driving force (concentration difference) for CO_2_ capture using the medium of Figure 1a. Additionally, from Figure 1b, it is clear that to enable CO_2_ capture from air, the pH should be at least 9.8, below which off‐gassing of CO_2_ could occur (shown as a negative driving force; Vadlamani et al., 2019). Because of the role of hydroxide ions in CO_2_ absorption, the absorption rate will increase exponentially with pH. During the mathematical modeling of CO_2_ mass transfer, the latter relationship is part of the enhancement factor (Figure 1c). It is clear that for effective air capture, the pH should be as high as possible.

Recent studies (Beardall, Wangikar, Mikulic, Vonshak, & Varshney, 2014; Engler et al., 2016; Hansen, 2002; Sharp et al., 2017; Vadlamani et al., 2019; Vadlamani, Viamajala, Pendyala, & Varanasi, 2017) have explored algae and cyanobacteria cultivation at a pH of up to 10.2, so still fall short of the requirements for effective air capture. Microalgal growth at high pH is challenging because of decreasing bicarbonate concentrations. As the pH increases above 10, biological energy requirements to run carbon concentrating mechanisms will increase. Carbon concentrating mechanisms act on bicarbonate, no biological mechanism is known for direct cellular import of carbonate. At these high pH values, carbon dioxide itself is no longer a relevant substrate because of its extremely low concentration.

Here, we demonstrate the combination of algal photosynthesis with effective air capture of CO_2_ at pH values between 10.5 and 11.2. We used microbial mats collected from alkaline soda lakes in British Colombia, Canada as a source of high pH and alkalinity adapted microorganisms. In nature, these mats contained abundant cyanobacteria growing at a (bi)carbonate alkalinity of 500–800 meq/L and pH of 10.1‐10.3. The mats were previously shown to yield robust and productive consortia of cyanobacteria after enrichment in photobioreactors at lower pH, up to 10 (Sharp et al., 2017). The present study shows that higher pH values enable effective regeneration of spent growth medium by air capture and, surprisingly, do not compromise biomass productivity.

## 2 EXPERIMENTAL METHODS

### 2.1 Photobioreactor cultivation setup

Cyanobacterial consortium previously enriched (using full spectrum, “white” light) from benthic microbial mats collected from Soda Lakes located on the Cariboo Plateau, British Columbia (Sharp et al., 2017) were grown as biofilms in acrylic tubular bioreactors (ground area = 29 cm^2^; See photograph in Figure S1). A green mesh was used as a scaffold for biofilm formation inside the bioreactors. A detailed schematic of the bioreactor is provided in the supplementary information (Figure S2). A synthetic growth medium contained: K_2_HPO_4_ (1.44 mM), NH_4_Cl (0.92 mM), NaNO_3_ (3.06 mM) MgSO_4_·7H_2_O (1 mM), CaCl_2_·2H_2_O (0.17 mM), KCl (6 mM), NaCl (0.43 mM), ferric ammonium citrate (10 mg·l^−1^) and 1 ml of trace metal solution. NaHCO_3_ and Na_2_CO_3_ concentrations were added according to initial pH requirement. A total of 4 L of growth medium was circulated through these tubular bioreactors at a flow rate of 10 ml·min^−1^. All the experiments were carried out at room temperature (~25°C). The tubular bioreactors were illuminated with two full spectrum led lights (Model T5H0; 6400K, Sunblaster Holdings ULC, Langley, BC, Canada), located on opposing sides of the bioreactors and a 16:8 light to dark photoperiod was maintained. The light intensity of ~150 μmol·m^−2^·s^−1^ was measured using a PAR sensor (Licor Biosciences, Lincoln, NE). Samples taken periodically and were analyzed to determine pH, total alkalinity (TA) and soluble nitrogen. Biofilms were harvested when there was either minimal amounts or no soluble nitrogen present in the growth medium.

### 2.2 Analytical methods

Ash free dry weight (for both inoculum and harvested biomass), TA and soluble nitrate were measured using previously reported methods (Vadlamani et al., 2017; Van Wychen & Laurens, [Link]). pH of the medium was measured periodically using a pH meter (Mettler‐Toledo, Columbus, OH). pH probe was calibrated daily over a pH range of 4–12. Ash free dry weight measurements were used to estimate the biomass productivity (Sharp et al., 2017). Once the growth cycle was completed, the accumulated biomass was manually scraped and washed off the green mesh, until no visible biomass remained. TA and pH of the growth medium were used to determine the bicarbonate and carbonate concentrations (Vadlamani et al., 2017). Biomass was also freeze dried using a freeze dryer (Labconco, NJ) and was used for elemental analysis.

### 2.3 Fluorometric measurements

A closed Fluorcam FC 800‐C (Photon System Instruments, Czech Republic) was used to measure the photosynthetic parameters. Cultivations were carried out at a smaller scale (in 20 ml petri dishes) by maintaining similar growth conditions (as described above) and used for fluorometric analysis. In brief, biofilm grown in a 20 ml petri dish was placed in Fluorcam FC 800‐C chamber and incubated in the dark for 10 min to measure the minimum fluorescence in the dark adapted state (*f*_*o*_). Then the sample was subjected to saturating pulse of 4,000 μmol·m^−2^·s^−1^ to obtain a maximum fluorescence in the dark adapted state (*f*_*m*_). These fluorescence parameters *f*_*o*_ and *f*_*m*_ were used to estimate dark adapted quantum yields. Finally, electron transfer rates were determined at incremental actinic light intensities (ranging from 2.5 to 1,000 μmol·m^−2^·s^−1^).

## 3 RESULTS AND DISCUSSION

Petri dishes containing a 0.8 µm membrane filter were inoculated with a cyanobacterial consortium previously enriched from alkaline soda lakes at a pH of 8.3. This consortium was dominated by a filamentous cyanobacterium affiliated with *Phormidium*, and also contained >100 other different species (Sharp et al., 2017). During these incubations, the consortium formed a biofilm on the membrane filter.

To investigate the effect of pH on photosynthesis, two sets of petri dishes were inoculated. For the first set, the pH was 8.5 initially, with 460 mmol/L sodium bicarbonate and 20 mmol/L sodium carbonate. For the second set, the pH was 10.4 initially, with 35 mmol/L sodium bicarbonate and 230 mmol/L sodium carbonate. After 4 days, the pH had increased from 8.5 to 9.42 in the first set and from 10.43 to 11.46 in the second set. As shown in Figure 1a, equation (2), the algal carbon concentrating mechanism, combined with carbon dioxide fixation, leads to an increase in pH.

**Figure 2:**
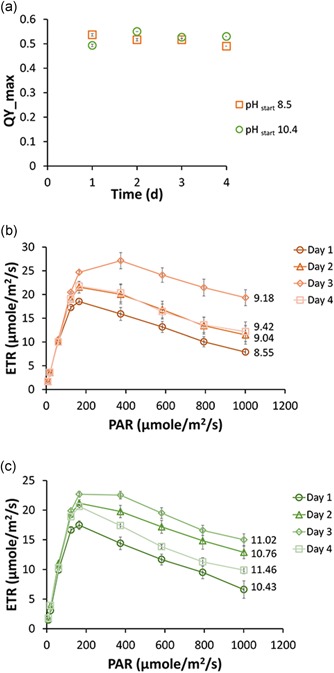
Effect of pH on photosynthetic activity of the Cyanobacterial consortium. (a) shows dark adapted quantum yield for cyanobacterial consortium grown in a starting pH of 8.5 (red) and 10.4 (green). (b,c) Showing electron transfer rates under different light intensities for 4 days of growth in lower pH (b) and higher pH (c). Numbers at the end of the lines indicate the pH measured on each day. Error bars indicate the standard deviation of six biofilm samples [Color figure can be viewed at wileyonlinelibrary.com]

During these experiments, the photosynthetic quantum yield and electron transfer rates were monitored off‐line using fluorescence imaging. Figure 2a shows that the quantum yield after dark adaptation was around 0.55 (55% efficient) during growth, independent of pH. This suggested that the photosystems were not negatively affected by the high pH and decreasing bicarbonate concentrations. Maximum electron transfer rates (Figure 2b,c) were also similar and independent of initial pH (18.5 μmol/m^2^/s at pH 8.3 vs. 17.5 μmol/m^2^/s at pH 10.4). As growth progressed and pH increased, electron transfer rates initially increased (up to 21.5 μmol/m^2^/s on the 2nd day) for both experiments and then decreased again. They did always remain above their initial values. Even though the bicarbonate concentration was much higher at pH 8.5, the electron transfer rates appeared to be independent of pH. This suggested that the rates of ATP and NAPDH turnover were similar, which could result in a similar carbon fixation rates. Because the carbon concentrating mechanism requires energy to operate, biomass yield would still be lower at high pH. Overall, fluorescence imaging of photosystem physiology indicated that photosynthesis functioned well, at least up to pH 11.46.

Next, we investigated the effect of pH on biomass productivity and nitrate and bicarbonate uptake rates. The cyanobacterial consortium was inoculated into replicated tubular photobioreactors (Figure S1), one set with initial pH 8.3, one set with initial pH 10.4. The photobioreactors were illuminated at an intensity of 150 µmol photons/m^2^/s for 16 hr per day. In both sets of experiments, the cyanobacterial consortium was observed to colonize the entire tubular photobioreactor within 4 days. No additional growth was observed after 4 days, presumably because the nitrogen sources (nitrate and ammonium) were fully depleted. After 4 days, there was still 256 ± 5 mmol/L of bicarbonate left in the first set. In second set, bicarbonate was almost fully consumed (<mmol/L). When nitrate was nearly depleted, the biomass was harvested, and a portion was used to start a new growth cycle. This way, three growth cycles (at low pH) and four growth cycles (at high pH) were performed.

**Figure 3:**
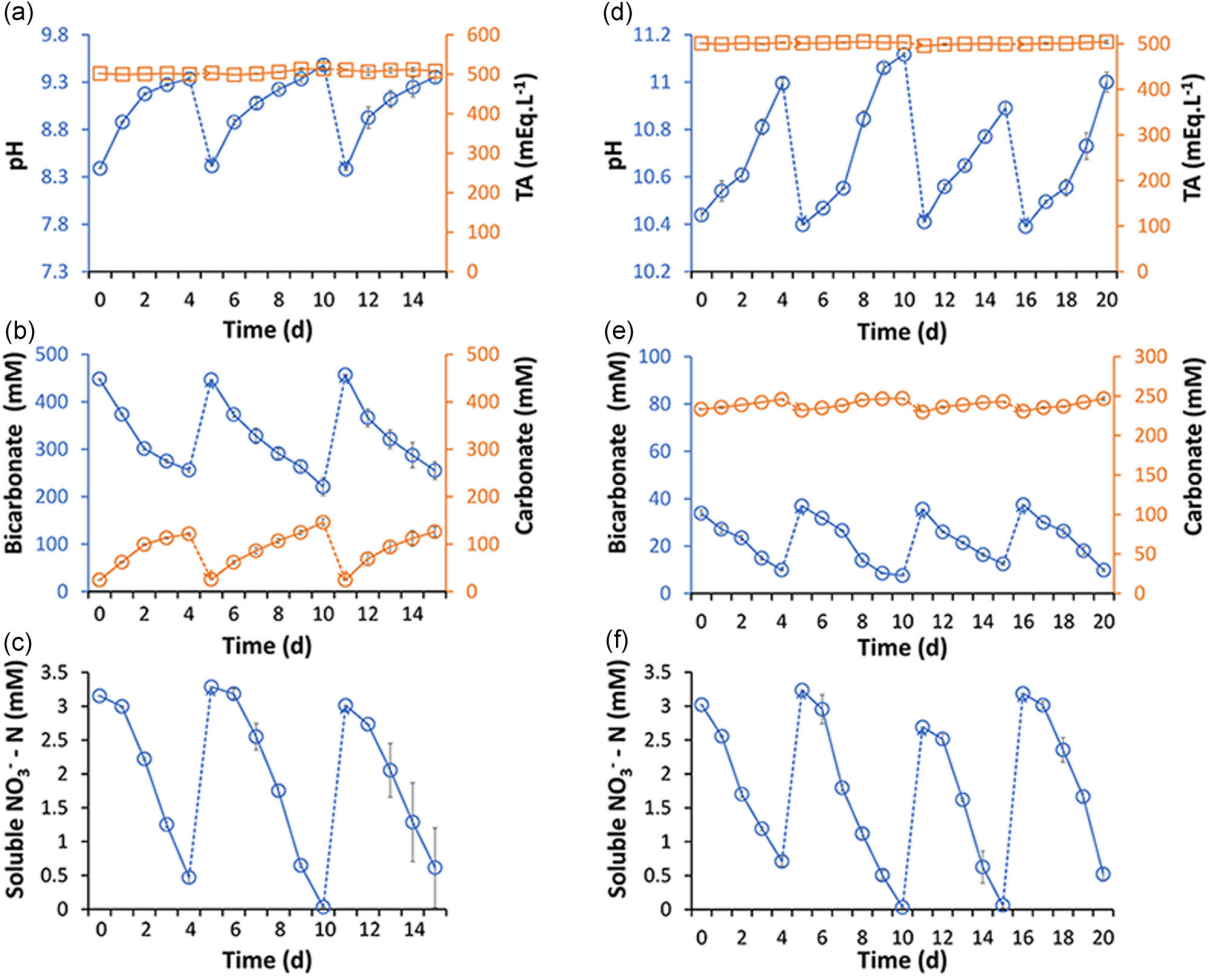
Effect of pH on nitrate and bicarbonate uptake rates. (a–c) Shows three growth cycles with initial pH of 8.3. (d–f) Shows four growth cycles with initial pH of 10.4. Each cycle was performed in duplicates and error bars indicate the standard deviation [Color figure can be viewed at wileyonlinelibrary.com]

**Figure 4:**
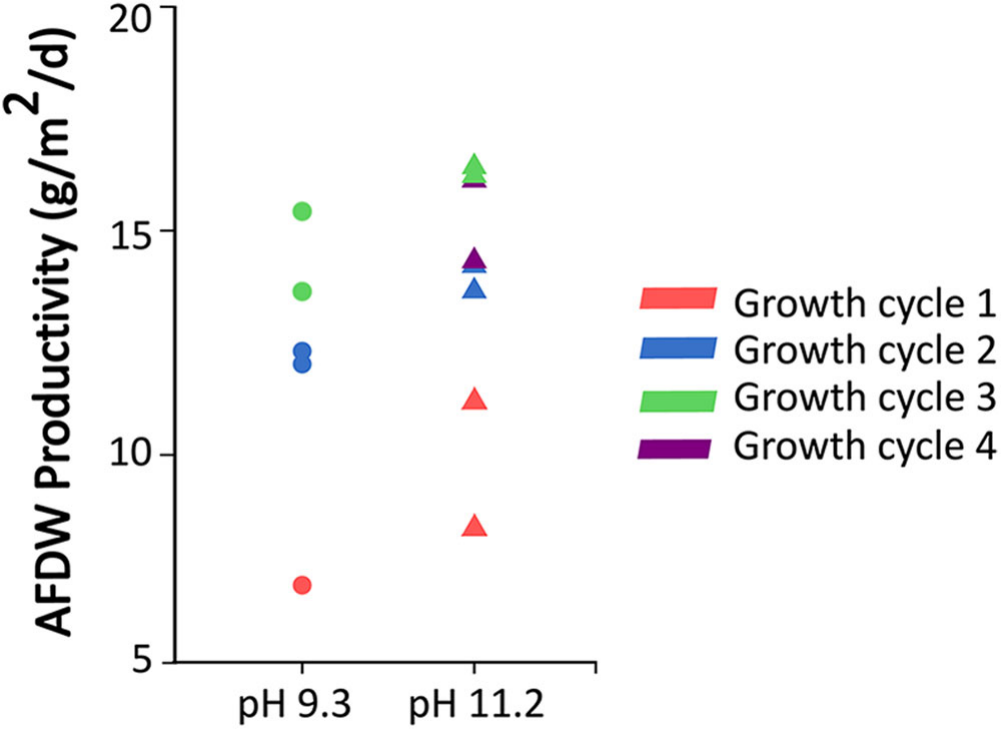
Effect of pH on biomass productivity of the system. Productivity is shown as ash free dry weight (AFDW) for bioreactors grown at pH 8.3–9.3 (shown as pH 9.3), and pH 10.5–11.2 (shown as pH 11.2) [Color figure can be viewed at wileyonlinelibrary.com]

In the low pH experiments, the pH increased from 8.3 to 9.3 (Figure 3a) during the first four‐day growth cycle. Bicarbonate and carbonate concentrations were monitored and showed the conversion of bicarbonate and production of carbonate (Figure 3b). In addition, 2.7 mmol/L of nitrate was used (Figure 3c). The uptake of bicarbonate and nitrate by the cyanobacterial consortium resulted in an overall increase in biomass. The estimated biomass productivity, based on ash free dry weight and photobioreactor illuminated surface area, was 7.1 g/m^2^/d. The outcomes of the second and third growth cycles were very similar across replicate experiments. However, the biomass productivity was found to increase with each harvest, up to 14.5 ± 0.9 g/m^2^/d after the third and last cycle (Figure 4). The increase in biomass productivity might be explained by ecological or physiological acclimatization of the cyanobacterial consortium to the experimental setup.

On average, 206.5 ± 17.4 mmol/L of bicarbonate disappeared from the medium during all three growth cycles, and 106.7 ± 12.2 mmol/L of carbonate was produced, consistent with equation (2). The difference would correspond to the uptake of 41.3 ± 2.2 g‐C/m^2^/d of carbon dioxide. However, the organic carbon accumulated in the biomass (estimated from CHN analysis and differences in ash‐free dry weight) amounted to only 4.8 ± 1.4 g‐C/m^2^/d. That means that 89% of the bicarbonate concentration decrease remained unaccounted for. Most likely, the missing bicarbonate escaped to the atmosphere during the experiment. Because the pH in these experiments remained below 9.3, there was always a driving force for CO_2_ transfer from the medium to the atmosphere, instead of vice versa (see Figure 1b). The growth medium was circulated through the tubular photobioreactors and a medium vessel, which was in contact with the atmosphere.

In the high pH experiments, the pH increased from 10.4 to 11.0 (Figure 3d) during the first 4‐day growth cycle. Bicarbonate and carbonate concentrations were monitored and showed the conversion of 23 mmol/L of bicarbonate and the production of carbonate (Figure 3e). The pH increase and bicarbonate and nitrate utilization rates were very similar for the subsequent three growth cycles (Figure 3d–f). As observed in the low pH experiments, the productivity increased with each growth cycle. The biomass productivity during the fourth growth cycle was 15.2 ± 1.0 g/m^2^/d, a 38% increase compared to first growth cycle (Figure 4). Again, the increase in productivity might be explained by the acclimatization of the cyanobacterial consortium to the experimental setup. Additionally, we have carried out a statistical analysis to determine the significance of the biomass productivities obtained over the three cultivation cycles. This analysis showed that the p‐value was significantly lower than alpha, which means that the biomass productivities obtained are statistically different.

The average bicarbonate depletion during growth cycles was 23.1 ± 7.1 mmol/L and 12.6 ± 4.1 mmol/L of carbonate was produced. The cyanobacterial consortium might have consumed the remainder, 11.0 ± 3.2 mmol/L for growth. This would be equivalent to an average carbon dioxide uptake rate of 4.6 ± 1.3 g‐C/m^2^/d. Interestingly, the organic carbon accumulated in the biomass (estimated from CHN analysis and differences in ash‐free dry weight) amounted to 7.7 ± 2.2 g‐C/m^2^/d, almost twice as much. Because the pH in these experiments was always above 10.4, there was a driving force for transfer of carbon dioxide from the air into medium (Figure 1b). Thus, the additional productivity might be explained by air capture during each growth cycle. The biomass productivity values were in the same range or higher than previously reported for alkaliphilic microalgal cultures (Table 1).

**Table 1:** Comparison of cultivation conditions and biomass productivity of the cyanobacterial consortium with previously reported literature for high pH growth systems

Cultivation strain	Bioreactor	pH	Alkalinity (M)	N Source	Light Intensity (µmol./m^2^/s)	T (°C)	AFDW (g/m^2^/d)	Reference
*Euhalothece* ZM001^a^	250 ml flasks	10.3	1	NO_3_^−^	100	35	2.1	Chi et al., 2013
*Euhalothece* ZM001^a^	250 ml flasks	10.3	1	Urea	100	35	1.1	Chi et al., 2013
*Euhalothece* ZM001^a^	250 ml flasks	10.3	1	NH_4_^+^	100	35	0.0	Chi et al., 2013
*Euhalothece* ZM001^a^	250 ml flasks	10.3	1	NH_4_^+^, NO_3_^−^	100	35	0.7	Chi et al., 2013
*Euhalothece* ZM001^a^	T‐flask	9.5	1	NO_3_^−^	250	35	6.9	Chi et al., 2013
*Neochloris oleoabundans*	Flatpanel	10	1.21	NO_3_^−^	50	25	11.7	Santos et al., 2013
Cyanobactira consortium^b^, ^c^	Flatpanel	10	0.5	NH_4_^+^	80	25	1.9	Sharp et al., 2017
Cyanobactira consortium^b^, ^d^	Flatpanel	10	0.5	NH_4_^+^	80	25	3.0	Sharp et al., 2017
Cyanobactira consortium^b^, ^e^	Flatpanel	10	0.5	NH_4_^+^	80	25	1.1	Sharp et al., 2017
Cyanobactira consortium^b^, ^d^	Flatpanel	9	0.5	NH_4_^+^	80	25	3.0	Sharp et al., 2017
*Chlorella sorkiniana* SLA‐04^a^	3 L Cytostir	10	0.012	NO_3_^−^	294	20	5.0	Vadlamani et al., 2017
*Arthrospira platensis* NIES39^a^	500 ml flasks	9.8	0.23	NO_3_^−^	160	35	13.0	Kishi and Toda, 2018
*Dunaliella salina* NIES2257^a^	500 ml flasks	9	0.5	NO_3_^−^	160	20	3.8	Kishi and Toda, 2018
*Euhalothece Sp.* ZM001^a^	500 ml flasks	10	1.1	NO_3_^−^	160	35	14.1	Kishi and Toda, 2018
Cyanobactira consortium^b^	Tubular	10.5	0.5	NO_3_^−^	150	25	16	Current study

*Note*. AFDW: ash free dry weight.

^a^ Cultures were incubated with 24 hr light intensity.

^b^ Cultures were incubated with 16:8 light/dark cycle.

^c^ Cultures were incubated using a white light source.

^d^ Cultures were incubated using a red light source.

^e^ Cultures were incubated using a blue light source.

**Figure 5:**
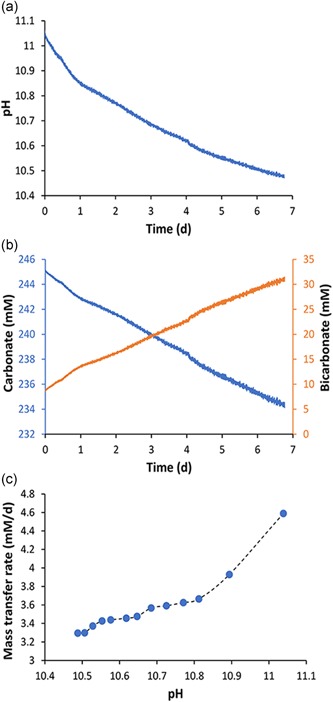
Effect of CO_2_ absorption from air on spent medium. (a) Shows change in pH while bubbling air into the medium. (b) Showing modeled changes in bicarbonate and carbonate concentrations during 7 days of air bubbling. (c) Showing estimated absorption rates of CO_2_ into the spent medium [Color figure can be viewed at wileyonlinelibrary.com]

Next, the possibility to use the spent medium (pH > 11) for air capture was investigated experimentally. Since, the purpose of our study was to replenish the depleted HCO_3_^−^ via CO_2_ capture from air, we now performed carbon capture experiments using air and spent growth media. Spent media (pH = 11.06), obtained after harvesting the biomass, was sparged (bubbled) with air for 7 days, with continuous pH measurement. Figure 5 shows the pH decrease and the modeled bicarbonate and carbonate concentrations, as well as the carbon dioxide capture rate. As expected, (Figure 1), the capture rate increased with pH. The slight increase observed between pH 10.5 and 10.8 was consistent with a slight increase in driving force (Figure 1b). The steeper increase above pH 10.8 was consistent with an exponential increase in hydroxide ion concentration (improved enhancement factor; Figure 1c). This experiment showed that spent medium used for growing cyanobacteria at high pH can be regenerated using CO_2_ capture from air. In practice, this would require an air‐scrubbing capture unit similar to power plant cooling towers, as previously proposed (Keith, Holmes, Angelo, & Heidel, 2018).

## 4 CONCLUSION

This study showed that an alkaliphilic cyanobacterial consortium obtained from an alkaline soda lake was able to grow at high pH (up to 11.2) and alkalinity (~500 mM). The biomass productivity was higher or comparable to previously reported values for alkaliphilic microalgal cultures. The pH swing during the algal growth cycle (10.5–11.2) enabled effective regeneration of the growth medium by direct CO_2_ capture from air.

## Supporting information

Supporting information
