# Supplementary material for: Direct capture and conversion of CO2 from air by growing a cyanobacterial consortium at pH up to 11.2
Source: Biotechnol Bioeng. 2019 Apr 8;116(7):1604–11. doi: 10.1002/bit.26974 (PMC6593468; doi:10.1002/bit.26974)
Supplement: Supplementary file 1 — Supporting information [file BIT-116-1604-s001.docx]

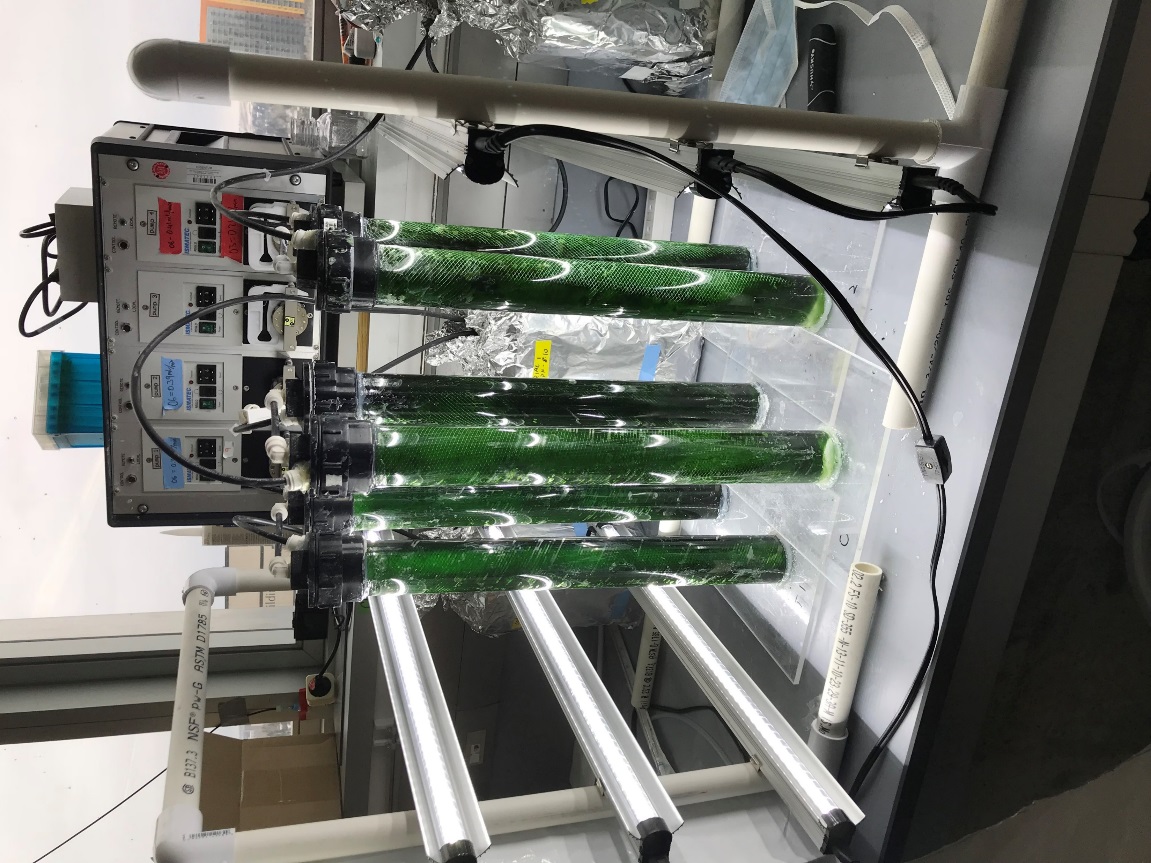


Figure S1: Photograph of the experimental set up of the tubular bioreactors


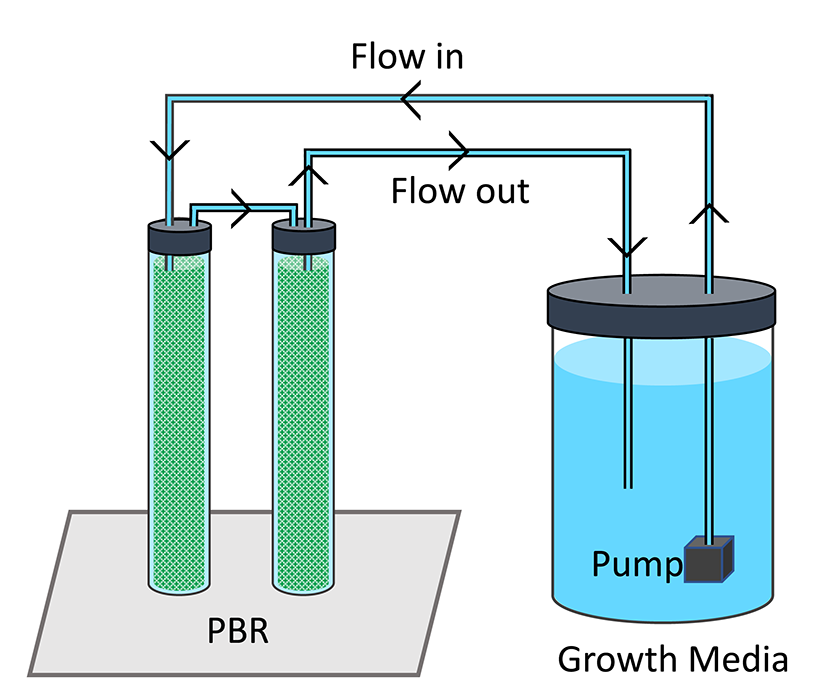


Figure S2: Schematic of the experimental set up of the bioreactors
